# Estrogen Receptor 1 Gene (*ESR1*) rs2234693 Polymorphism and Breast Cancer Risk in Saudi Women

**DOI:** 10.31557/APJCP.2020.21.11.3235

**Published:** 2020-11

**Authors:** Razan Jamaan Al-Amri, Mohammad Kdaimes H Alotibi, Rawya Ibrahim AL-Raddadi, Weam Talal Yahya Shebli, Emad Ibrahim Yagoub Fallatah, Ahmed Safar Alhujaily, Hiba Salaheldin Mohamed

**Affiliations:** 1 *Department of Biology, College of Science, Taibah University, Madinah, Kingdom of Saudi. *; 2 *Department of Oncology, King Fahad Hospital, Madinah, Kingdom of Saudi Arabia. *; 3 *Department of Pathology, King Fahad Hospital, Madinah, Kingdom of Saudi Arabia. *; 4 *Institute of Endemic Diseases, University of Khartoum, Sudan. *

**Keywords:** Genetic variant, ESR1, breast cancer, Saudi Arabia

## Abstract

**Objective:**

The present study aimed to determine the role of *ESR1* gene rs2234693 T/C polymorphism (PvuII) in the susceptibility to breast cancer and to assess the association of this polymorphism within presence or absence of estrogen, progesterone receptors, human epidermal growth factor receptor 2 (HER2) and with premenopausal and postmenopausal age in Saudi women.

**Methods:**

The study was a retrospective case-control study. In this study, 137 breast cancer and 98 normal breast paraffin embedded tissues were included. DNA was extracted and *ESR1* gene rs2234693 T/C polymorphism was genotyped by PCR-RFLP. Genetic association tests were performed.

**Results:**

The results showed no significant difference in distribution of rs2234693 T/C alleles and genotypes frequencies. Odd ratios (95% CI) were 1.15 (0.8-1.66) and 1.06 (0.5-1.98) and p values were 0.51 and 0.87, respectively. The genotypes and alleles frequencies within different hormonal receptors groups and ages of menopause showed no signification association (odd ratios were less or close to 1 and p values > 0.05).

**Conclusion:**

*ESR1* gene rs2234693 T/C polymorphism was not associated with susceptibility to breast cancer and different menopausal, hormone receptors, and HER2 status in breast cancer patients. Further analysis using larger sample size will be needed to assess the association of different polymorphisms within the gene and risk of breast cancer.

## Introduction

Breast cancer (BC) is a serious health issue, causing changes within the body cells and out of control cell grow (Alotaibi et al., 2018; Saggu et al., 2015). Saudi Cancer Registry (SCR) recorded a total of 6,922 female BC cases between January 2001 and December 2008. The number of female BC cases was 1,152, 1,308, and 1,473 in 2008, 2009, and 2010, respectively. In 2010, BC ranked first (5,378 cases, 27.4%) among females with newly diagnosed cancers (Saggu et al., 2015; Saudi Cancer Registry, 2005). In 2018, IARC reported BC as the most prevalent cancer in Saudi Arabia with 3629 new cases out of all other cancers among women (https://gco.iarc.fr/today/data/factsheets/populations/682-saudi-arabia-fact-sheets.pdf.). 

Studies have shown that BC is a complex disease and factors such as gender, age, ethnicity, family history, genetic factors, menopausal status, radiation exposure, alcohol, and exposure to high dose of estrogen are associated with the risk of developing BC (Nindrea et al., 2017). Effects of estrogen are mediated through two distinct nuclear receptors, namely estrogen receptor alpha (ERα) encoded by *ESR1* gene and estrogen receptor beta (ERβ) encoded by *ESR2* gene (Deroo and Korach, 2006). Estrogen and its receptor ER1 have major function in development and progression of cancer. ER1 is an important mediators of hormonal response and a target for BC hormonal treatments (Debeb and Berihu, 2015). It also stimulates mammary epithelial tissue proliferation and differentiation through combining with estrogen (Clemons and Goss, 2001). *ESR1* gene, located on chromosome 6q25.1, consists of eight exons and seven introns (Ponglikitmongkol et al.,1988). *ESR1* gene is a low-penetrance BC susceptibility gene. It may increase the likelihood of accumulation of genetic mutations occurring throughout cellular division. It functions as a ligand and stimulates cell proliferation and controls cell growth and cell death by binding to both endogenous and exogenous hormones. Genome-wide association studies have identified several single nucleotide polymorphisms (SNPs) within *ESR1* gene that are associated with predisposition to BC. *ESR1* rs2234693 (PvuII) polymorphism is located in intron one and it is one of the most characterized SNPs of* ESR1*gene (Hill et al.,1989). It is also associated with increased risk of BC and other diseases in which estrogen is implicated (Deroo and Korach, 2006). Possible purposeful mechanism attributed to the present polymorphism includes a change in the expression of the *ESR1* gene by altering the transcription factor binding sites and affecting alternative splicing of the *ESR1* gene. However, the allele variant has been shown to be associated with BC risk in different populations (Chauhan et al., 2019). The present study aimed to find out the possible role of *ESR1* gene rs2234693 T/C polymorphism in the susceptibility to BC. We also tried to evaluate the association of this polymorphism within presence or absence of estrogen, progesterone receptors, and human epidermal growth factor receptor 2 (HER2) and menopausal status.

## Materials and Methods

*Study design and samples*

Breast tissue samples of Saudi women were collected from King’s Fahad Hospital. All samples were archive, antonyms, and paraffin-embedded (FFPE )tissues. Clinical data of each patient was retrieved from King Fahad Hospital database. This hospital-based case-control study included 137 histopathological confirmed BC cases and 98 benign tissue samples as controls. The cases and controls were matched in age. Samples with different BC subtypes, including different receptors status ER, PR, HER2, and triple-negative (ER-, PR-, HER2-), were included.

*DNA extraction and genotyping*

The DNA was extracted from 5 to 8 pieces of 10 μm thickness sections of paraffin embedded tissue . Through incorporation of several steps of heating and shaking, modified phenol-chloroform protocol method was used.

The primers were designed to amplify *ESR1* gene rs2234693 (T/C) polymorphism. Forward primer was 5’ GGGTTATGTGGCAATGACGT 3’, and reverse primer was 5’ GACCAATGCTCATCCCAACTC 3’ (Macrogen Company, South Korea) with an expected product size of 164 bp. The amplification was performed using T100™ Thermal Cycler (Bio-Rad, Germany) instrument in a total volume of 25 µL by Maxime PCR PreMix Kit (Intron biotechnology, South Korea). The PCR mix consisted of 2.5mM dNTPs, 5U/µl i-TaqTM polymerase, 1X reaction buffer, gel loading buffer, 1µl of 10mM of each primer, and 1µl of template DNA (100ng/ µl). PCR conditions consisted of 35 cycles following a hot start at 95ºC for 3 minutes. Each cycle included the following three steps: DNA denaturation (30 second at 94ºC), primer annealing (30 second at 62ºC), and primer extension (1 minute at 72ºC). There was a final extension cycle for 5 minutes at 72ºC. The size of the amplified product was determined using 1% agarose gel electrophoresis. The amplified PCR product was digested by the PvuII enzyme (Thermo Fisher Scientific, USA). The DNA fragments were separated using 3% low melting agarose gel. Restriction digestion by PvuII produced two fragments of 108 and 56bp.

*Sequencing*

Six samples were selected from three different genotypes (Homozygous wild type T/T, Heterozygous T/C, and Homozygous mutant C/C). PCR was performed in volume of 50µl and sent to Macrogen (Korea) company for sequence analysis, using same forward and reverse primers, to confirm RFLP genotype results. 

*Genetic statistical analysis*

Allele and genotype frequencies of PvuII polymorphism of the genotypes were calculated. Deviation from Hardy Weinberg equilibrium was tested. Genetic association tests were performed using AssociatORRR software (https://www.genecalculators.net/associatorrr-cc.html). Odd ratio (OR) with 95% confidence intervals (CI) was used to express association. P-value <0.05 was considered statistically significant. 

## Results

*Demographic information and clinicopathological features of of BC patients*

The average age of our patients was 51.2±14 and 59 (43.1%) ones aged < 50 and 70 (51.1%) aged ≥50. Invasive-infiltrating ductal carcinoma was the most frequent histological subtype in our patients (78.1%). Most of the patients were diagnosed at advanced clinical grades of II and III, 40.9% and 40.1%, respectively. The results on hormone receptor status showed that 74 (54%) out of 119 patients were ER-positive, 60 (43.8%) were PR-positive, and 38 (27.7%) were HER2-positive. Molecular subtyping using surrogate immunohistochemistry (IHC) markers showed that luminal A subtype predominated in patients (43.8%), followed by HER2-like (15.3%), and luminal B (12.4%), while triple-negative subtype was represented in 13.9% of the patients.

*RCR-RFLP*

**Figure 1:**
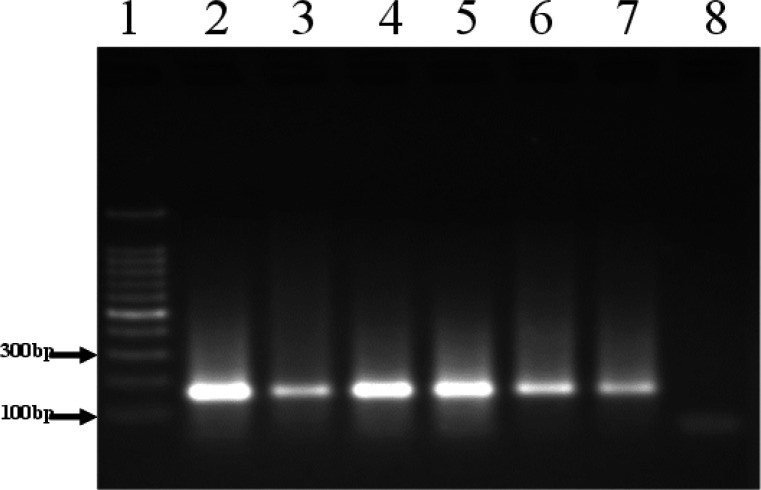
Representatives gel for PCR product. Lane 1, represents 100bp DNA molecular weight marker; Lane 2 to 7, PCR product (164bp); Lane 8, negative control

**Figure 2:**
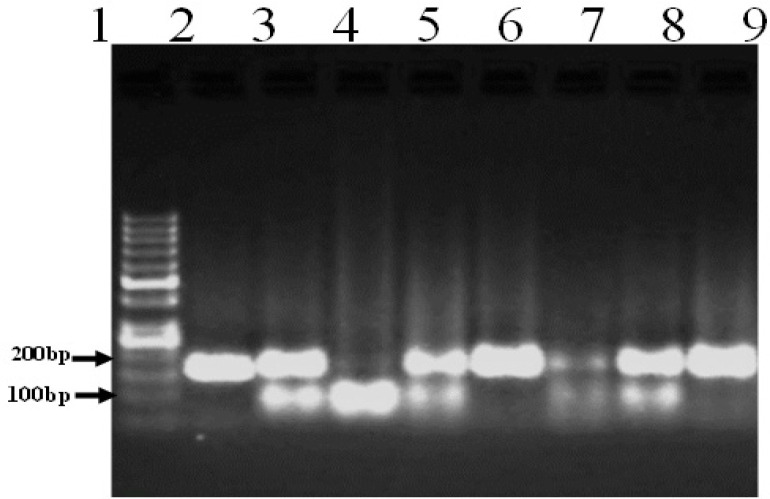
Representative gel for Ggenotyping of *ESR1* Gene Polymorphism. Lane 1, Represents 50bp DNA molecular weight marker; Lane 2, PCR product undigested (164bp); Lane 3 ,5, 7 and 8, Heterozygous T/C (164,108,56 bp). Lanes 6 and 9: Homozygous mutant C/C (164bp); Lane 4, Homozygous wild type T/T (108,56 bp)

The PCR product for *ESR1* gene rs2234693 T/C polymorphism generated size band of 164bp (Figure 1). Genotyping using the PvuII enzyme showed three different patterns (Figure 2).

*Sequencing*

**Figure 3:**
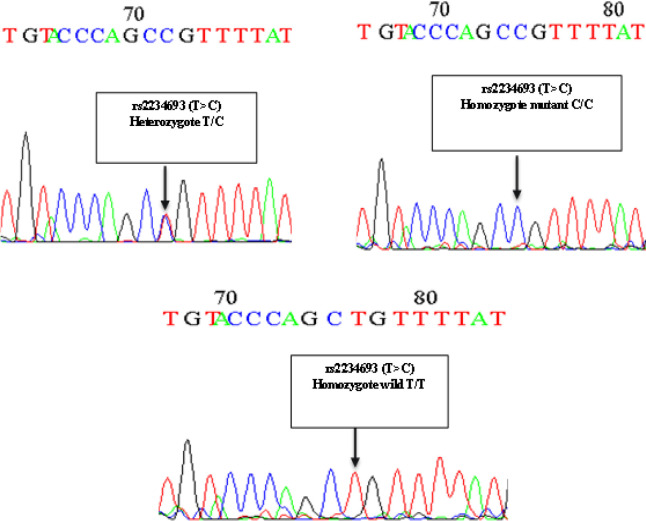
Electropherogram of Three Different Genotypes

The sequencing results obtained from Macrogen (Korea) company confirmed our results on different genotypes (Figure 3).

*Genetic statistical analysis*

The genetic analysis demonstrated similar distribution of PvuII polymorphism alleles and genotypes frequency between controls and cases (Table 1). The genotype frequencies revealed 0.24 of wild homozygous (TT), 0.49 of heterozygous (TC), and 0.27 of mutant homozygous (CC) in the cases. In the control group, the genotype frequencies were 0.27 of wild homozygous (TT), 0.51 of heterozygous (TC), and 0.22 of mutant homozygous (CC). The Minor allele frequencies were (C allele) 0.51 and 0.48 in cases and controls, respectively. There was no significant association between *ESR1* gene rs2234693T > C polymorphism and susceptibility to BC. Table 2 depicts a summary on the distribution of genotypes by menopausal status and hormonal receptor status. The results did not suggest any genetic association between menopausal status and predisposition to BC. No association was detected between *ESR1* gene rs2234693 within presence or absence of the estrogen and progesterone hormones and HER2 in tumor samples (Table 2). The genotype and allele frequencies were in Hardy–Weinberg equilibrium where p values were >0.05. 

**Table 1:** Genotype Frequencies of *ESR1* Gene rs2234693T/C Polymorphism in Breast Cancer Patients versus Controls

Allele/Genotype	Cases (frequency) n = 137	Controls (frequency) n = 98	OR (95%Cl)	P* value
T	133 (0.49)	102 (0.52)	1.15 (0.8-1.66)	0.51
C	141 (0.51)	94 (0.48)		
T/T	33 (0.24)	26 (0.27)	1.06 (0.56-1.98)	0.87
T/C	67 (0.49)	50 (0.51)		
C/C	37 (0.27)	22 (0.22)		

*ESR1*, Estrogen receptor1 gene; OR, odd ratio (OR≤1 or close to 1 means the SNP does not affect odds of disease outcome); Cl, Confidence interval; n, Total number of samples; P* value, 2 tailed P value- Fisher's Exact Test. Cut off ≤ 0.05

**Table 2:** Polymorphism of *ESR1* PvuII and Breast Cancer Risk by a Menopausal Status and Receptors Statues

Genotype/allele	Controls (frequency) n=98	ER+ cases n=74	ER− cases n=45	Controls vs. ER+ OR (95%CI) P* value	Controls vs. ER− OR (95%CI) P* value	
T	102 (0.52)	79 (0.53)	44 (0.49)	0.95 (0.62~1.45)	1.13 (0.69~1.87)	
C	94 (0.48)	69 (0.47)	46 (0.51)	p = 0.83	p = 0.7	
TT	26 (0.27)	22 (0.3)	10 (0.22)	0.83 (0.41-1.7)	1.25 (0.52-3)	
TC	50 (0.51)	35 (0.47)	24 (0.53)	p = 0.87	p = 0.67	
CC	22 (0.22)	17 (0.23)	11 (0.24)			
Genotype/allele	Controls (frequency) n= 98	PR+ Cases n=60	PR− cases n=58	Controls vs. PR+ OR (95%CI) P* value	Controls vs. PR− OR (95%CI) P* value	
T	102 (0.52)	66 (0.55)	56 (0.48)	0.89 (0.56~1.40)	1.16 (0.73~1.84)	
C	94 (0.48)	54 (0.45)	60 (0.52)	p = 0.64	p = 0.56	
TT	26 (0.27)	20 (0.33)	12 (0.20)	0.68 (0.32-1.43)	1.39 (0.61-3.1)	
TC	50 (0.51)	26 (0.43)	32 (0.55)	p = 0.34	p = 0.54	
CC	22 (0.22)	14 (0.23)	14 (0.24)			
Genotype/allele	Controls (frequency) N=98	HER+ cases n=38	HER− cases n=80	Controls vs. HER+ OR (95%CI) P* value	Controls vs. HER− OR (95%CI) P* value	
T	102 (0.52)	38 (0.50)	84 (0.53)	1.09 (0.64~1.84)	0.98 (0.65~1.49)	
C	94 (0.48)	38 (0.50)	76 (0.48)	p = 0.79	p = 1	
TT	26 (0.27)	11 (0.29)	21 (0.26)	0.67 (0.31-1.86)	1.04 (0.51-2.1)	
TC	50 (0.51)	16 (0.42)	42 (0.53)	p = 0.64	p = 1	
CC	22 (0.22)	11 (0.29)	17 (0.21)			
Genotype/allele	Controls (frequency) n=98		TNBC cases n=19		Controls vs. TNBC OR (95%CI) P* value	
T	102 (0.52)		18 (0.47)		1.21 (0.60~2.42)	
C	94 (0.48)		20 (0.53)		p = 0.72	
TT	26 (0.27)		3 (0.16)		2.08 (0.54-8.03)	
TC	50 (0.51)		12 (0.63)		p = 0.37	
CC	22 (0.22)		4 (0.21			
Genotype/allele	Pre controls n=74	Pre cases n=59	Post controls n=15	Post cases n=71	Controls vs. Pre OR (95%CI) P* value	Controls vs. Post OR (95%CI) P* value
T	79 (0.53)	55 (0.47)	14 (0.47)	73 (0.51)	1.31 (0.81~2.13)	0.83 (0.38~1.82)
C	69 (0.47)	63 (0.53)	16 (0.53)	69 (0.49)	p = 0.32	p = 0.69
TT	21 (0.28)	13 (0.22)	3 (0.20)	19 (0.27)	1.27 (0.54-2.05)	0.69 (0.16-2.91)
TC	37 (0.50)	29 (0.49)	8 (0.53)	35 (0.49)	p = 0.68	p = 0.74
CC	16 (0.21)	17 (0.29)	4 (0.27)	17 (0.24)		

*ESR1,* Estrogen receptor1 gen; ER, Estrogen receptor; PR, Progesterone receptor; HER2, Human epidermal receptor 2 (HER2); TNBC,Triple negative breast cancer; Pre, Premenopausal; Post, Postmenopausal; N, number of samples; OR, odd ratio. (OR≤1 or close to 1 means the SNP does not affect odds of disease outcome); CI, confidence interval; P* value, 2 tailed P value- Fisher’s Exact Test. Cut off ≤ 0.05

## Discussion

BC is a multifactorial disease and it is not yet fully understood. GWAS has shown that the fundamental BC mechanisms are composed of genetic and environmental factors, and certain SNPs within susceptibility genes may have an impact on the development of BC (Albalawi et al., 2019). Numerous clinical studies have investigated SNPs within *ESR1* gene while the most widely studied polymorphisms of *ESR1* is rs2234693 (Abd Ellatif et al., 2016). This was the first study conducted in KSA investigating *ESR1* gene PvuII polymorphism and BC. The changes in *ESR1* gene expression are possible functional mechanisms attributed to PvuII polymorphism (Anghel et al., 2010). It was reported that substitution of T by C led to loss of PvuII restriction site , and the resulted C allele produced myb transcription factor binding site, significantly increasing transcription compared to the T allele (Herrington et al., 2002). Several diseases, including BC (Zhang et al., 2015; Zhang et al., 2018), endometrial cancer (Ashton et al., 2009), prostate cancer (Li et al.,,2017), Alzheimer’s disease (Cheng et al., 2014), endometriosis and leiomyoma (Hsieh et al., 2007), and hepatocellular and gallbladder cancer (Sun et al., 2015) were assessed for probable association with PvuII polymorphism. In this study, no genetic association was found between PvuII polymorphism and BC risk. Similar results were shown in different populations, including Korean, Swedish, Americans, and Portugal (Shin et al., 2003; Sonestedt et al., 2009; Alves et al., 2010; Clendenen et al., 2013). The PvuII polymorphism of the *ESR1* gene was associated with susceptibility to BC in different population such us Chinese, Indian, and Egyptian (Cai et al., 2003; Abd Ellatif et al., 2016; Chauhan et al., 2019). There are contrasting results in this regard. Some studies demonstrated that *ESR1* gene PvuII polymorphism was significantly associated with the lower risk of BC in Asians but not in Caucasians populations ( Zhang et al., 2018). In a Meta-analysis, it was yielded that CC genotype was associated with an elevated risk of BC. However, TT or CT genotypes increased this risk in European and Caucasian (Hu et al., 2017). In different studies, PvuII ‘C’ allele was associated with high risk of BC in Asians, but with no risk in Euro-Americans population (Lu et al., 2013). This findings indicate that ethnicity and geographic location can contribute to the breast carcinogenesis (Zhang et al., 2015). The results of stratified analysis in this study did not reveal any genetic association between menopausal, hormonal, and growth receptors status and susceptibility to BC. Different studies indicated that menopausal status had an effect on estrogen production, cell signaling, and metabolism (Li and Xu, 2012). Several meta-analysis studies indicated that the *ESR1* PvuII polymorphism was associated with high risk of BC in pre-menopausal in comparison with post-menopausal women (Cai et al., 2003; González-Zuloeta Ladd et al., 2008). However, many studies (Shin et al., 2003; González-Mancha et al., 2008; Anghel et al., 2010), including this study, did not show any genetic associations between menopausal status and increased risk of BC. 

The PvuII polymorphism was associated with ER expression in BC patients (Hill et al., 1989). In another study, ER status was not associated with susceptibility to BC, and PvuII C allele was found in high frequency in patients with PR negative (Anderson et al., 1994). This association was not found in the present study. Nevertheless, our results were in agreement with many studies in different populations, including Caucasian, Chinse, and Portugal populations ( Cai et al., 2003; Alves et al., 2010; Anghel et al., 2010). 

In conclusion, these results indicated that *ESR1* 2234693 T/C polymorphism was not associated with susceptibility to BC in Saudi women. It is likely that ethnic variation and environmental influences among different populations have an effect on association between SNPs and possible risk of BC. Further studies are needed to confirm this finding and assess the association of other polymorphisms within *ESR1* gene with risk of BC .

*Ethic statement*

All study procedures were agreed by Taibah University, College of Dentistry Research Ethics Committee (TUCDREC/20170607), and from the Department Research Committee at King Fahd Hospital (ADCARE), Madinah
